# Regularity and mechanism of fake crackle noise in an electronic stethoscope

**DOI:** 10.3389/fphys.2022.1079468

**Published:** 2022-12-12

**Authors:** Peitao Ye, Qiasheng Li, Wenhua Jian, Shuyi Liu, Lunfang Tan, Wenya Chen, Dongying Zhang, Jinping Zheng

**Affiliations:** ^1^ National Center for Respiratory Medicine, State Key Laboratory of Respiratory Disease, National Clinical Research Center for Respiratory Disease, Guangzhou Institute of Respiratory Health, First Affiliated Hospital of Guangzhou Medical University, Guangzhou, China; ^2^ Faculty of Medicine, Macau University of Science and Technology, Macau, China

**Keywords:** fake crackle, crackle, electronic stethoscope, auscultation, frequency

## Abstract

**Background:** Electronic stethoscopes are widely used for cardiopulmonary auscultation; their audio recordings are used for the intelligent recognition of cardiopulmonary sounds. However, they generate noise similar to a crackle during use, significantly interfering with clinical diagnosis. This paper will discuss the causes, characteristics, and occurrence rules of the fake crackle and establish a reference for improving the reliability of the electronic stethoscope in lung auscultation.

**Methods:** A total of 56 participants with healthy lungs (no underlying pulmonary disease, no recent respiratory symptoms, and no adventitious lung sound, as confirmed by an acoustic stethoscope) were enrolled in this study. A 30-s audio recording was recorded from each of the nine locations of the larynx and lungs of each participant with a 3M Littmann 3200 electronic stethoscope, and the audio was output in diaphragm mode and auscultated by the clinician. The doctor identified the fake crackles and analyzed their frequency spectrum. High-pass and low-pass filters were used to detect the frequency distribution of the fake crackles. Finally, the fake crackle was artificially regenerated to explore its causes.

**Results:** A total of 500 audio recordings were included in the study, with 61 fake crackle audio recordings. Fake crackles were found predominantly in the lower lung. There were significant differences between lower lung and larynx (*p* < 0.001), lower lung and upper lung (*p* = 0.005), lower lung and middle lung (*p* = 0.005), and lower lung and infrascapular region (*p* = 0.027). Furthermore, more than 90% of fake crackles appeared in the inspiratory phase, similar to fine crackles, significantly interfering with clinical diagnosis. The spectral analysis revealed that the frequency range of fake crackles was approximately 250–1950 Hz. The fake crackle was generated when the diaphragm of the electronic stethoscope left the skin slightly but not completely.

**Conclusion:** Fake crackles are most likely to be heard when using an electronic stethoscope to auscultate bilateral lower lungs, and the frequency of a fake crackle is close to that of a crackle, likely affecting the clinician’s diagnosis.

## 1 Introduction

Auscultation is a standard physical examination method used by physicians and is widely accepted by doctors and patients because of its simplicity, repeatability, and non-invasiveness. Auscultation can be classified as direct or indirect. Direct auscultation attaches the ear directly to the body wall of the examined person, while indirect auscultation uses a stethoscope. Because the sound heard by direct auscultation is weak and inconvenient to operate, indirect auscultation has gradually replaced direct auscultation since the invention of acoustic stethoscope (Sarkar et al., 2015).

With its low price and ease of use, the acoustic stethoscope is still the most common auscultation tool despite 200 years of evolution and development (Vyshedskiy et al., 2009; Bank et al., 2016). However, several limitations of the acoustic stethoscope are evident, such as interference from environmental noise, low volume, and the lack of storage and playback functions. Given these defects, the electronic stethoscope emerged (Tavel, 2006; Bank et al., 2016; Rennoll et al., 2021). In 1956, Airsonic Limited (London) produced a new electronic stethoscope that included a miniature battery-operated amplifier, a contact microphone, and conventional ear-pieces. It overcame the poor sound quality of previous electronic stethoscopes and had a good environmental noise reduction effect. The stethoscope only produced noise when its diaphragm was in contact with the body (Taylor and Fothergill, 1956). So far, there is no report that the modern electronic stethoscope can filter this noise, even though it has undergone some evolution and improvement.

Nowadays, the application of electronic stethoscope in cardiopulmonary sound auscultation is increasing gradually. Further, their audio recording characteristics are used for intelligent recognition of cardiopulmonary sounds. However, noise similar to a fine crackle, referred to as a *fake crackle*, is generated during use, which may interfere with the doctors’ clinical diagnosis of patients. A crackle is a discontinuous, explosive, non-musical adventitious lung sound (Murphy, 1981; Sarkar et al., 2015), with a frequency between 200 and 1200 Hz. It is often detected in the respiratory phase because it is generated by the sudden opening of the airway during inspiration (Forgacs et al., 1971; Forgacs, 1978; Vyshedskiy et al., 2009). Crackles are commonly observed in chronic obstructive pulmonary disease, bronchiectasis, pneumonia, pulmonary edema, and other respiratory diseases, as well as heart failures (Bettencourt et al., 1994; Piirilä and Sovijärvi, 1995; Tilkian and Conover, 2009; Bohadana et al., 2014). Crackles can be roughly divided into coarse crackles and fine crackles. The coarse crackles have a loud intensity and low pitch, with a duration of about 15 m and a typical frequency of about 350 Hz. The fine crackles have a quieter intensity and a higher pitch, with a duration of about 5 m and a typical frequency of about 650 Hz (Bohadana et al., 2014; Sarkar et al., 2015).

At the end of the 20th century, some scholars explored the automatic detection of lung sounds (Kaisla et al., 1991; Sovijärvi et al., 1998; Vannuccini et al., 1998). Due to the separation of the recording process from the auscultation process, the collection efficiency of the lung sounds is low, and the sample size is too small for research. Hence, the resulting lung sound recognition model is not widely used.

The electronic stethoscope can simultaneously collect lung sounds and stethoscope, which simplifies the collection process of lung sounds. Therefore, some studies on intelligent lung sound recognition have used an electronic stethoscope, such as the 3M Littmann 3200, to collect data (Grzywalski et al., 2019; Kevat et al., 2020; Hsu et al., 2021; Nguyen and Pernkopf, 2022). However, the recorded lung sounds still need to be heard by annotators and labeled manually before they can be used in the training algorithm. The fake crackles may be labeled as crackles, affecting the crackle feature extraction and the recognition rate of the pulmonary sound classification model. This study aims to reveal the causes, characteristics, and occurrence rules of fake crackles and establish a reference for improving the reliability of the electronic stethoscope during pulmonary auscultation.

## 2 Methods

### 2.1 Study population

We recruited 56 participants (29 males and 27 females) with good pulmonary status from the First Affiliated Hospital of Guangzhou Medical University. All participants were Chinese, aged between 20 and 35 years old and their Body Mass Indexes (BMIs) was within the normal range, excluding those with underlying pulmonary disease, recent respiratory symptoms, or adventitious lung sounds detectable by acoustic stethoscopes. This study was approved by the Ethics Committee of the First Affiliated Hospital of Guangzhou Medical University (Approval number: 2017-82), and all participants signed a written informed consent.

### 2.2 Data collection

**FIGURE 1:**
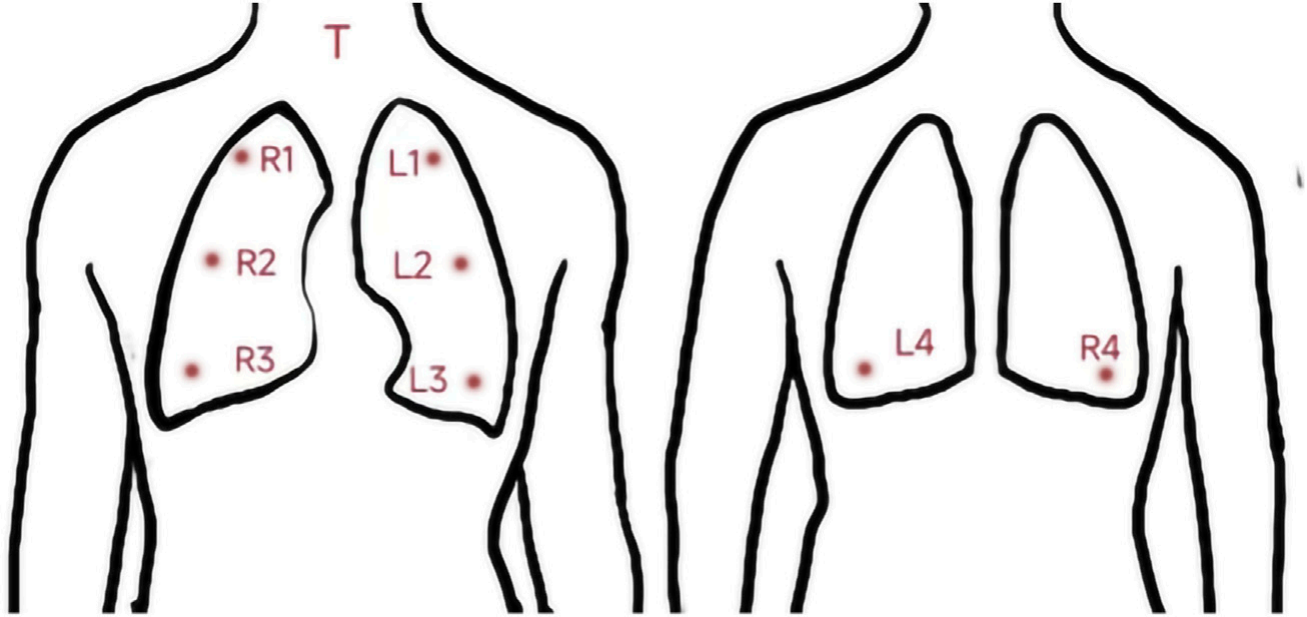
Position of the 9 auscultation locations. T: Throat (Larynx); RI: Right upper lung; R2: Right middle lung; R3: Right lower lung: R4: Right infrascapular region; L1: Left upper lung: L2: Left middle lung; L3: Left lower lung; L4: Left infrascapular region.

Respiratory sounds were recorded using a 3M Littmann 3200 electronic stethoscope with a sampling rate of 4000 Hz and a bit depth of 16 bits. All audio recordings were recorded in a quiet environment (below 40 dB), and the stethoscope’s diaphragm was close to the skin of chest wall during recording. Respiratory sounds of nine locations (denoted as T, R1–R4, and L1–L4) were collected successively. The auscultation locations are described in detail in the caption of Figure 1. Each location recorded 30 s of audio, and each participant recorded nine audio recordings. If the breath sounds were too weak, the participant was instructed to breathe more deeply so that the breath sounds could be heard clearly by the human ear. “Littmann StethAssist” software was used to connect the stethoscope to a computer immediately after each participant was recorded and the data were transferred to the computer. The data were then output in diaphragm mode to WAVE (.wav) format and named for the location (e.g., T or L1).

### 2.3 Spectral analysis

**FIGURE 2:**
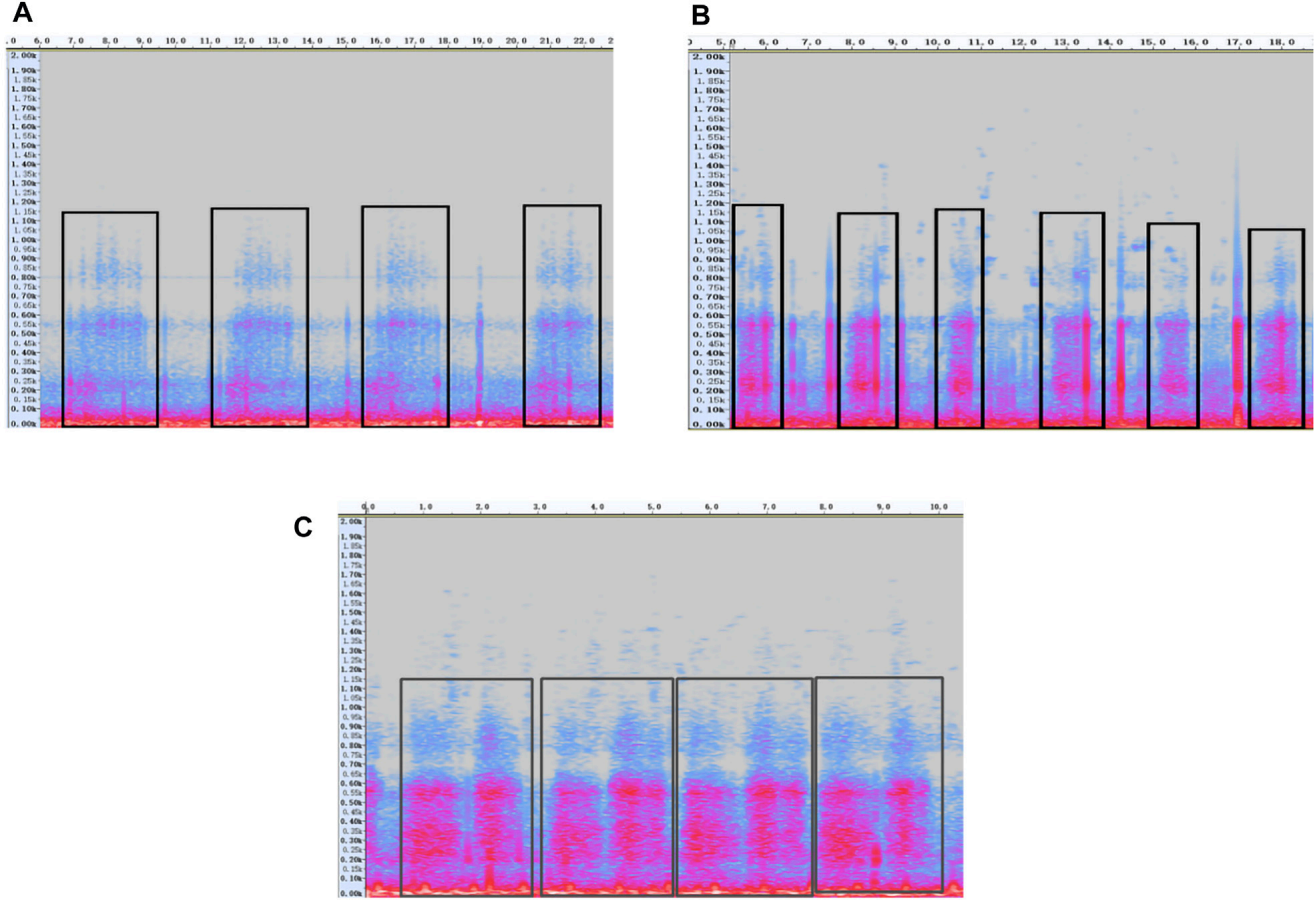
Example of the spectrogram of fake crackle, crackle and normal breath sound. The purple-pink areas are where the sound signals are, the vertical axis is frequency (Hz), and the horizontal axis is time (s). **(A)** Fake crackle, inside the black rectangles are the fake crackle regions; **(B)** Crackle, in the black rectangles are the crackle regions; **(C)** Normal breath sound, each black box is a breathing cycle.

“Audacity” software was used to open the audio recordings and select the spectrogram mode (Figure 2). The respiratory physician listened to each audio recording one by one. If a fake crackle was heard in an audio recording, then high-pass and low-pass filters were used to gradually lock the frequency of the fake crackle. When the filter was not filtered thoroughly, the spectrum selection function was enabled to delete the frequency band to be filtered. We used the determination of the minimum frequency as an example.

The spectrogram of the fake crackle in Figure 2A reveals that the maximum frequency was approximately 1200 Hz. We selected the 1200–2000 Hz frequency band, deleted it, and listened to the audio carefully. If the fake crackle could still be heard, we selected the 1150–1200 Hz frequency band, deleted it, and again listened to the audio carefully. The frequency band of 50 Hz was deleted more each time than the previous one, and the listening process was repeated until the fake crackle could no longer be heard. Then, the maximum frequency after the last frequency band deletion was the minimum frequency of the fake crackle in this audio recording. A similar method was used to determine the maximum frequency. Only the frequency band below 1200 Hz needed to be deleted, followed by repeating the process of listening and deleting the spectrum.

### 2.4 Statistical analysis

All statistical analyses were performed using SPSS 25 statistical software. GraghPad Prism 9.0 and Microsoft Office 2019 were used for the plotting. The frequency of the fake crackle was statistically described by the mean and standard deviation. Pearson’s chi-squared test was used to analyze the difference between fake crackles generated by electronic stethoscope auscultation in males and females. Cochran’s Q test was used to compare the statistical difference of fake crackles generated by electronic stethoscope auscultation in different locations, with a statistically significant *p*-value of less than 0.05.

## 3 Results

Of the 56 participants in the study (29 males and 27 females), 34 heard a fake crackle when they were auscultated with an electronic stethoscope. We collected 504 audio recordings from 56 participants, of which 4 were excluded because of operating errors that caused the duration to be less than 5 s or respiratory sounds not being recorded. The remaining 500 were included in the study.

Fake crackles were found in 61 of 500 recordings, of which 56 (91.80% of the total number of fake crackle audio recordings, posterior same) were heard in the inspiratory phase, 2 (3.28%) in the expiratory phase, and 3 (4.92%) in the respiratory biphasic phase. There were 261 audio recordings for men, 33 of which had fake crackles, and 239 audio recordings for women, 28 of which had fake crackles. There was no significant difference in fake crackles generated using an electronic stethoscope in males and females (*p* = 0.751).

**FIGURE 3:**
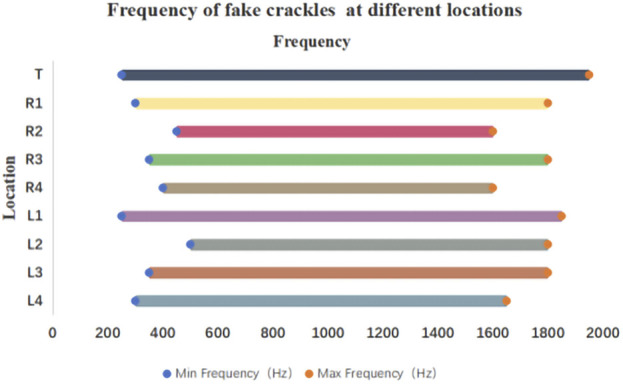
Frequency of fake crackles at different locations. As can be seen in the figure, the common frequency band of fake crackles in all locations is 500–1600 Hz.

The minimum and maximum frequencies of fake crackles in each audio recording are presented in Supplementary Table S1. After spectrum analysis of each audio recording with a fake crackle, we found that the minimum frequency of the fake crackle was between 250 and 1150 Hz, the maximum frequency band was 1050–1950 Hz, the middle frequency band was 700–1375 Hz, and the mean middle frequency was 1052.05 ± 99.40 Hz (1 standard deviation). The frequencies of fake crackles at different locations are depicted in Figure 3. Although they differed slightly, there was a common frequency band for all locations of 500–1600 Hz.

**FIGURE 4:**
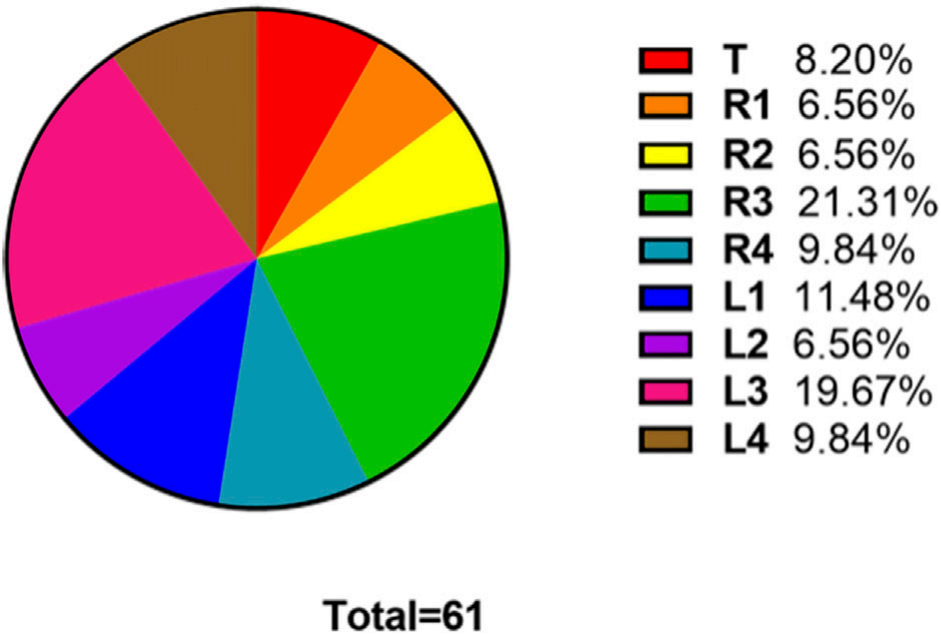
The ratio of the number of fake crackles audio recordings at each location to the total number of fake crackles audio recordings. The figure shows that the number of fake crackles audio recordings in R3 and L3 are significantly more than that in other locations.

**FIGURE 5:**
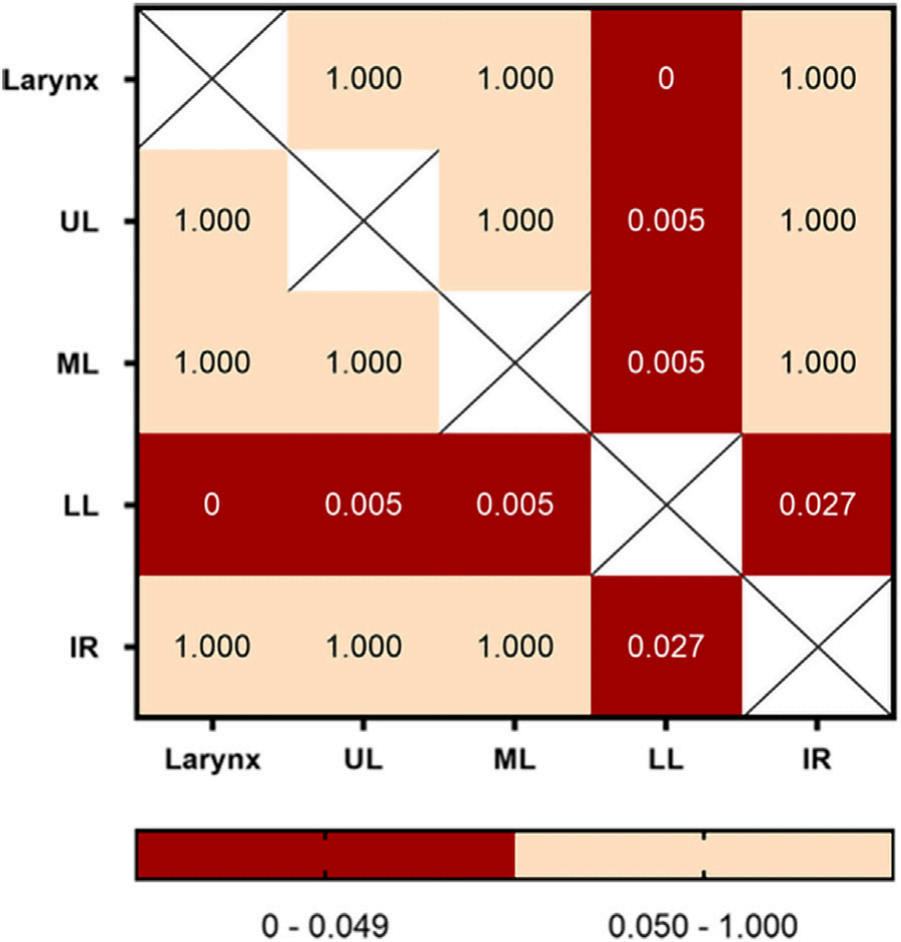
Pairwise comparison of fake crackles in different locations. The number in each cell is the *p*-value of the comparison between the two locations, and combinations with *p*-values less than 0.05 are shown in red. Abbreviation: UL: upper lung; ML: middle lung; LL: lower lung; IR: infrascapular region.

The numbers of fake crackle audio recordings in each auscultation location are presented in Table 1 and account for the total number of fake crackle audio recordings, as depicted in Figure 4. The left lower lung (L3) and right lower lung (R3) have the most fake crackles, accounting for 19.67% and 21.31% of the total number of fake crackle audio recordings. There was no significant difference in fake crackles between the nine auscultation locations (*p* = 0.061). We then combined L1 and R1 into the upper lung, middle lung (R2 and L2), lower lung (R3 and L3), and infrascapular region (R4 and L4). The overall difference between fake crackles at the five locations was significant (*p* < 0.001). A pairwise comparison of each location revealed significant differences between lower lung and larynx (*p* < 0.001), lower lung and upper lung (*p* = 0.005), lower lung and middle lung (*p* = 0.005), and lower lung and infrascapular region (*p* = 0.027). In contrast, there was no significant difference in the pairwise comparison of other locations. The results of the pairwise comparison of each location are depicted in Figure 5.

**TABLE 1:** Distribution of fake crackles in each location.

Location	The number of fake crackles audio recordings N = 500	
	Have (%)	No (%)
T	5 (8.93%)	51 (91.07%)
R1	4 (7.14%)	52 (92.86%)
R2	4 (7.27%)	51 (92.73%)
R3	13 (23.21%)	43 (76.79%)
R4	6 (10.71%)	50 (89.29%)
L1	7 (12.96%)	47 (87.04%)
L2	4 (7.27%)	51 (92.73%)
L3	12 (21.43%)	44 (78.57%)
L4	6 (10.71%)	50 (89.29%)
Total	61 (12.20%)	439 (87.80%)

## 4 Discussion

### 4.1 Introduction of fake crackle concept

In this observational study, a noise similar to a fine crackle can occur when auscultation is performed with the 3M Littmann 3200 electronic stethoscope, referred to as a *fake crackle*. Fake crackles could be heard in all lung auscultation locations. There was no significant difference between males and females, and the fake crackles were most common in the lower lungs.

### 4.2 Spectrum analysis of fake crackles

Based on a spectrum analysis of fake crackles, their frequencies were between 250 and 1950 Hz, while the typical frequency of a fine crackle is approximately 650 Hz (Munakata et al., 1991). The frequency of a fine crackle is within the frequency range of a fake crackle, and it is difficult for human ears to distinguish between them. Figure 2 shows the spectrogram of fake crackles, crackles and normal breath sounds. It is found that fake crackles and crackles are discontinuous sounds, while normal breath sounds are continuous sounds. In a study of intelligent lung sound recognition by Hsu et al. (Hsu et al., 2021), the F1 score (the harmonic mean of precision and recall) of a crackle was only about 50%; more than 40% of the audio used in the training algorithm was collected using a 3M Littmann 3200 electronic stethoscope. We surmise that some of the fake crackles were labeled as crackles, resulting in poor feature extraction of crackles and a reduced recognition rate.

### 4.3 Analysis of causes of fake crackles

In attempting to regenerate a fake crackle artificially, we found that the diaphragm of the electronic stethoscope would generate fake crackle noise when it left the skin slightly but not entirely. During inspiration, the thorax expanded outward, and the thoracic expansion of bilateral lower lungs was the largest. The stethoscope’s diaphragm then passively dislocates with the thoracic expansion, resulting in a fake crackle. This phenomenon explained why the fake crackle noise was distributed predominantly in the lower lung and inspiratory phase. Electronic stethoscopes can amplify lung sounds tens of times more than acoustic stethoscopes, but this may also amplify the noise generated by the electronic stethoscope, interfering with the doctor’s assessment of what is happening in the patient’s lung.

### 4.4 Limitations of the study

The population included in this study was mainly young people, with a low probability of chronic respiratory diseases, normal BMIs, low abnormal fat accumulations in the chest wall, and clear respiratory audio displays. The limitation of this study is that the pulmonary imaging examinations of the participants were not completed to rule out organic lesions in the lungs. Instead, the participants were judged to have good respiratory conditions based on their self-reported absence of underlying pulmonary diseases, recent respiratory symptoms, and no adventitious sounds during bilateral lung auscultation. Pulmonary infectious diseases are generally prone to producing audible crackles in the bilateral lower lungs (Bettencourt et al., 1994; Vyshedskiy et al., 2011), and fake crackles in this study were most common in the bilateral lower lungs. Therefore, the possibility that few patients with mild lung disease were included in this study cannot be ruled out. Furthermore, we only used the audio recorded by one type of electronic stethoscope for the analysis. However, we tested several types of electronic stethoscopes and found fake crackles in all of them. Due to the small sample size collected and the lack of a standardized collection of lung sounds in various locations, they were not included in this study. Theoretically, the cause and occurrence rule of fake crackle generation by different types of electronic stethoscopes are the same. Unfortunately, this study did not identify a suitable method to eliminate fake crackles. However, the spectral characteristics and causes of fake crackles identified by this study can serve as a reference for subsequent product development.

## 5 Conclusion

Our study proposed (to the best of our knowledge) for the first time that fake crackles may be generated by an electronic stethoscope during auscultation. In this study, we researched the cases and occurrence rules of fake crackles and analyzed their frequency spectrum. Our findings suggest that fake crackles are most likely to be heard when using an electronic stethoscope to auscultate the bilateral lower lungs and that the frequency of a fake crackle overlaps with a crackle, reducing the reliability of electronic stethoscopes during pulmonary auscultation.

## Data Availability

The datasets presented in this article are not readily available because the original contributions presented in the study are included in the article/Supplementary Material, further inquiries can be directed to the corresponding authors. Requests to access the datasets should be directed to DZ, 13480271412@163.com.
